# A hyperspectral vessel image registration method for blood oxygenation mapping

**DOI:** 10.1371/journal.pone.0178499

**Published:** 2017-06-01

**Authors:** Qian Wang, Qingli Li, Mei Zhou, Zhen Sun, Hongying Liu, Yiting Wang

**Affiliations:** 1 Shanghai Key Laboratory of Multidimensional Information Processing, East China Normal University, Shanghai, China; 2 Jinling Hosp, Dept of Tangshan branch/sanatorium, Nanjing University, Sch. Med., Nanjing, China; National University of Defense Technology College of Mechatronic Engineering and Automation, CHINA

## Abstract

Blood oxygenation mapping by the means of optical oximetry is of significant importance in clinical trials. This paper uses hyperspectral imaging technology to obtain in vivo images for blood oxygenation detection. The experiment involves dorsal skin fold window chamber preparation which was built on adult (8–10 weeks of age) female BALB/c nu/nu mice and in vivo image acquisition which was performed by hyperspectral imaging system. To get the accurate spatial and spectral information of targets, an automatic registration scheme is proposed. An adaptive feature detection method which combines the local threshold method and the level-set filter is presented to extract target vessels. A reliable feature matching algorithm with the correlative information inherent in hyperspectral images is used to kick out the outliers. Then, the registration images are used for blood oxygenation mapping. Registration evaluation results show that most of the false matches are removed and the smooth and concentrated spectra are obtained. This intensity invariant feature detection with outliers-removing feature matching proves to be effective in hyperspectral vessel image registration. Therefore, in vivo hyperspectral imaging system by the assistance of the proposed registration scheme provides a technique for blood oxygenation research.

## Introduction

The study of blood oxygenation proves to be fundamental in clinical trials involving oncology, clinicoroentgenologic, and phototherapy [1]. Considerable techniques have been created to support the researches in determination of blood oxygenation. Among these techniques, optical blood oximetry proves to be significant in providing a noninvasive assessment of oxygen saturation [2]. The feasibility of this optical oximetry emerges from the absorption, scattering, and reflectance properties of hemoglobin [3]. Moreover, the development of optical spectroscopy makes it possible to use in vivo imaging technique with high-resolution, real-time monitoring and accurate specificity analyzing methods [4]. To fully understand the mechanism of blood oxygen saturation at the microvascular level, one of the most essential models of in vivo imaging is dorsal skin fold window chamber (DSFC). The concept was first proposed by Algire, when he adapted it to the mouse for a preliminary trial [5]. Since then, DSFC has been applied in pathology because of its advantages in non-invasive, chronic intravital microscopy [6]. For example, K. S. Øye and his coworkers proposed a method of measuring velocity and direction of blood flow in tumors and normal tissues [7]. A. J. Moy et al. described a protocol for surgical implantation and wide-field functional imaging to obtain hemodynamic information [8]. These prior studies accelerated maturity of DSFC in different stages and verified practicality in analysis of hemopathology mechanism. Based on the model, consequently, in vivo imaging can acquire information through various image technologies, such as building sequential images into mathematical model [9], or applying the optical micro-angiography imaging system [10]. Although a number of methods have been developed to address relevant extraction from in vivo images, most of these researches mainly focus on spatial information rather than spectral information.

Hyperspectral imaging technology containing sufficient spectral information may provide reliable methods for in vivo images of oxygen saturation analysis. Theoretically, spectral signatures reflect the absorption of a certain object in a specific range of wavelength, usually from the ultraviolet (UV) to near-infrared (NIR). Thus, specific spectrum varies independently in different substance as long as the range broad enough. On the base of this characteristic, researchers can detect and analyze the essential components of complicated substances. Hyperspectral imaging technology has been applied to medicine for microscopic studies of anatomy, physiology, and hematology since 1990s [11], and good results have been achieved in several aspects, such as disease diagnose [12], mechanism study [13], and cancer detection [14]. These studies have shown that the combination of spatial and spectral information obtained by hyperspectral imaging system contributes to detection and diagnosis of pathological changes. As a result, some advanced studies combine hyperspectral imaging system with in vivo imaging for microvascular oxygen saturation analysis. B. S. Sorg et al. described their application of hyperspectral imaging for in vivo microvascular tumor oxygen transport studies using red fluorescent protein (RFP) and hypoxia-driven green fluorescent protein (GFP) [15]. M. C. Skala and his coworkers combined hyperspectral imaging with spectral domain optical coherence tomography (SDOCT) to monitor changes in hemoglobin saturation [16]. G. Hanna et al. established a system to visualize and quantify dynamic changes in hemoglobin saturation of lung function [17]. However, most of the existing studies rarely involved the registration problem stemmed from the conjoint hyperspectral in vivo imaging method.

The primary challenge when applying hyperspectral technology to in vivo detection is that for the same scene of hyperspectral image, pixels in one single band may shift to adjacent position in the next single band. This problem is common in *in vivo* imaging of DSFC because of continuous beating pulse of experimental animals. However, the hyperspectral imaging system needs to take quantative images in several seconds to obtain the spectrum of each pixel and the spectra must be integrated spatially in order to conduct spectral analysis, requiring the subject stay motionless. That is to say, under some circumstances the irregular twitch caused by in vivo imaging makes the pixels shift nonlinear so that a registration process instead of the simple alignment needs to be used to solve this problem. However, the primary objective of hyperspectral image registration methods commonly used in the remote sensing field is to geometrically overlay two images of the same scene (i.e., the reference and sensed images) taken at different times, from different viewpoints, or by different sensors [18]. Therefore, the commonly used hyperspectral image registration methods fall into three categories including multisource data fusion [19], multitemporal image changes [20] and image mosaicing [21]. These methods aim to register between different images rather than seeking correspondence within the same hyperspectral image. In order to obtain the accurate spectra of hemoglobin in hyperspectral in vivo image, the new registration algorithm need to be explored.

In general, there are mainly three steps to perform a registration algorithm: feature detection, feature matching and image transformation [22]. However, the chosen method of each step has its corresponding problems should be considered. Firstly, a proper feature detection method should be selected from two categories: area based and feature based. In area based methods, the principle of similarity is measured by a moving window which is set to calculate the densities between two images [23]. When the images have various changes in gray level, it’s hard to maintain its accuracy. Whereas, the feature based methods aim at extracting the specific feature points such as edges, boundaries and structures, which are invariant to scaling, rotation, and translation. In view of the hyperspectral images containing large quantities of pixels and its densities varying significantly, the feature based methods are the preferable choice in the feature detection step. Since the Speeded-Up Robust Features (SURF) algorithm was firstly proposed by H. Bay, it has been widely used in feature detection for its strong repeatability, distinctiveness, and robustness [24]. However, when applying the SURF method to hyperspectral images, a large number of redundant and false features increase difficulties for feature matching and image transformation. Thus, there comes to the second problem in the registration process. Generally, feature matching process means matching a group of template feature points to another group of image feature points [25]. The Random Sample Consensus (RANSAC) [26] algorithm is used to solve this problem for its advantage in finding the correct correspondences from the data containing a reasonable percentage of outliers. Nevertheless, the accuracy and stability is declining with the increase of outliers so that many research have been done to enhance resistance to the massive outliers, such as the combination of RANSAC and a Huber kernel [27], the fast sample consensus (FSC) [18] and prior energy function (P-RANSAC) [28]. Most of these studies mainly focus on the spatial information contained in a single image to find the correct matching points, whereas seldom concern the correlative information in the multispectral bands.

In this paper, the DSFC based in vivo imaging system was used to capture hyperspectral images of mice’s blood vessel. In order to utilize the spectral information for blood oxygen saturation analysis, we proposed a registration scheme specific for in vivo hyperspectral images. The threshold and level-set combined feature detection method was used to extract the target vessels from hyperspectral vessel images. Then, a correlative information based feature matching algorithm was presented and applied to modify the matching pairs. The experimental results show that most of the false matches were removed after registration process. Therefore, the clean and accurate spectrum of mice’s vessel can be obtained and used to perform the oxygen saturation analysis.

## Methods

**Fig 1:**
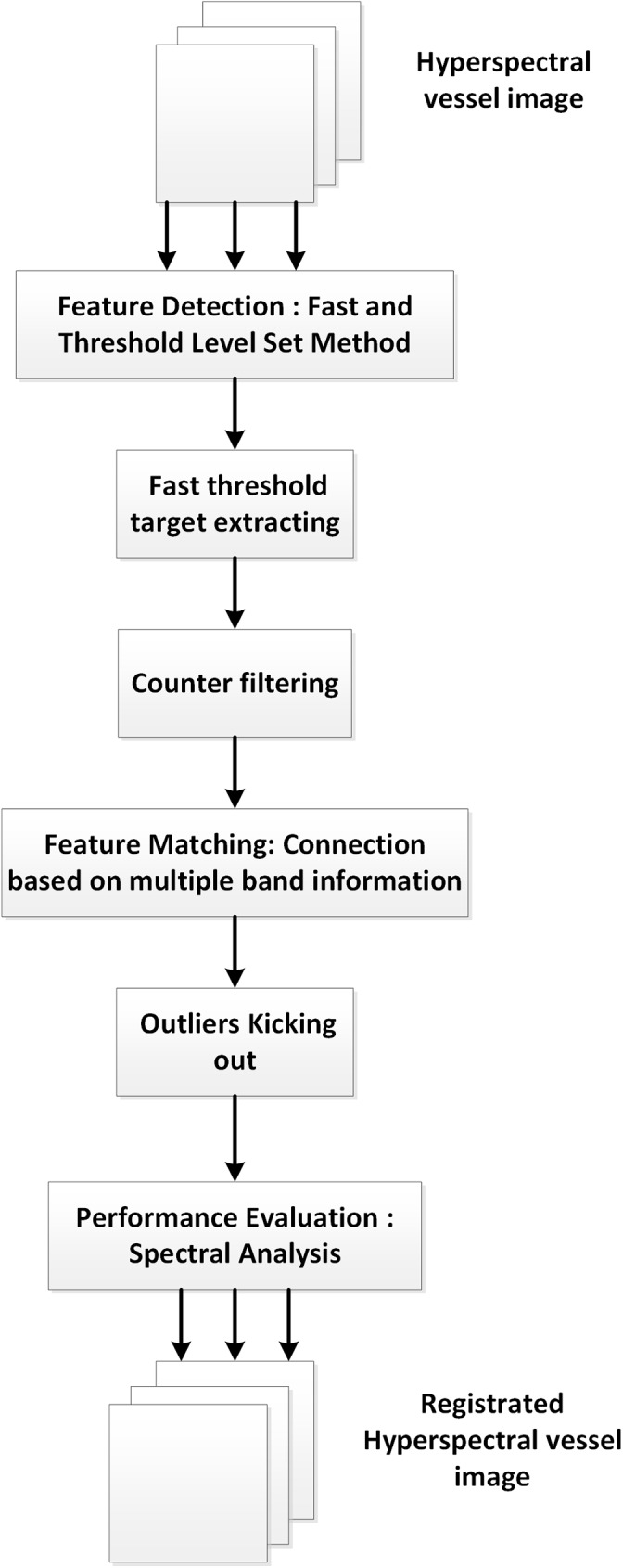
Flow of the proposed hyperspectral vessel image registration scheme.

As shown in Fig 1, substantial enhancement has been made for essential components of this proposed automatic registration process, including feature detection, feature matching and registration evaluation. Specific to in vivo hyperspectral vessel image, we focus on image segmentation in feature detection, outliers kicking out in feature matching and spectral analysis in registration evaluation. For image segmentation part, the even and continuous vessel target is obtained by determining a local threshold and constructing a level-set filter. After the influence of intensity variation among multiple bands is eliminated, the SURF algorithm is applied on the binary images to analyze the feature points. For image matching part, a reliable method is proposed based on correlative information among different bands to kicking out outliers. In spectral space, we use the spectral analysis to evaluate the performance of hyperspectral vessel images registration scheme.

### Feature detection

The purpose of feature detection is to find the key points of the target in the feature-based registration scheme. Therefore, the first step requires segmenting the target vessel from the complicated background and the second step extracting the feature points of the target vessel. As for the image segmentation part, the method depends on the type, content and characteristic of the image. In this study, we proposed a method that can separate the target vessel out of massive capillaries in hyperspectral vessel images. After obtaining the desirable counter of the vessel, the SURF algorithm is applied to feature extraction. SURF is a local feature detector and descriptor that have proven to be novel scale- and rotation-invariant, of which the abundant implementation and optimal results can be founded in [24, 29, 30]. However, the determination of segmentation methods considers more limitations and restrictions.

The first issue in hyperspectral images segmentation is that the spectral intensity of the same substance varied from band to band, which was induced by different light transmittance at different wavelengths. Therefore, the common principle for segmentation is not suitable for the spectrum intensity variation, so that we cannot apply the existing method on the hyperspectral images without adaptation. In order to eliminate the influence of multiple intensity variation, the algorithm based on thresholds is used to separate the target vessel from background. However, there are massive capillaries whose intensities are close to the main vessel so that this method may cause many noisy points and uneven blur. Compared with the former type, geometric deformable models which are based on the theory of front evolution and implemented using the level set numerical method, are more robust to noise and complicated counters [31, 32]. Geometric deformable models have been extensively applied to medical image processing with various applications [33, 34]. Starting from an initial counter, it will gradually track the target counter. However, this principle makes it sensitive to the initial counter and sometimes may cause boundary leaking.

To get over these weaknesses, we proposed a method named Fast and Threshold Level Set, which extracts the target vessel with even and continuous border and produces little noisy pixels. The method is composed of two processes: fast threshold target extracting and counter filtering. In the former process, we used threshold method to turn each band image into binary one, based on the idea of finding the optimal threshold that maximizes the between-class variance.

$$\sigma_{B}^{2}=\omega_{0}\omega_{1}{\left(\mu_{1}-\mu_{0}\right)}^{2}$$ (1)

where ω_0_ and ω_1_ denote the probabilities of class occurrence, μ_0_ and μ_1_ denote the mean gray level of the target vessel and the background, respectively. To avoid a large black area caused by obscure grey level difference between the two classes, we introduce a HsW (horizontal sliding window) for local application of the Otsu algorithm [35]. The Otsu method is a threshold based segmentation method which has been widely used in many areas [36, 37]. The criterion for transforming the original image into the binary one is as follows:

$$HsW(i,j)=\left\{ \begin{array}{l} LoT(HsW(i,j))\;if\;\sigma^{2}(HsW(i,j))\geq \sigma_{B}^{2}\\ 255\;if\;\sigma^{2}(HsW(i,j))\leq \sigma_{B}^{2} \end{array}\right.$$ (2)

where LoT() is the Otsu algorithm and *σ*^2^(*HsW*(*i*,*j*)) is the variance of pixels in the HsW. The target vessel can be fast extracted from hyperspectral images but the binary image is noisy and uneven. So a further step is needed to make the feature more significant.

In the counter filtering process, we smooth the target vessel from the noisy points by the level set method. Since the former process has detected the rough target vessel, the initial counter is determined correctly making the method more robust and faster. Then the speed function drive the moving counter iteratively until the terminate condition is reached. In this part, we choose geometric deformable model to calculate inner properties of the counter to control this speed function. Besides, the calculation is based on the binary image obtained from the first step, decreasing computational complexity significantly. Finally, the second process outputs the segmented vessel counter.

After the desirable counter is obtained from the segmentation part, it is facilitative for SURF method to detect feature descriptors and find correspondence among the vessels. However, the second issue came after we use the SURF to extract the feature points in each binary image. Although SURF performs better than the other detectors, there are still some incorrect matches, especially when it is applied to process the hyperspectral images, the false matches are up to thousands of pairs. Therefore, an effective feature matching method is needed to eliminate the false matches.

### Feature matching

After obtaining feature points from the feature detection part, the next requirement is to establish reliable correspondence between them. Although various feature matching methods have been proposed in different hyperspectral imaging fields, it is still a challenging task especially for the combined in vivo vessel images. First, a hyperspectral image contains numbers of bands and produces thousands of raw matches of feature points between every two bands. The large scale of feature points increase burden on the computational cost of common feature matching algorithms, whereas in vivo imaging stress on the effectiveness. Thus, to make the feature matching method more efficient, it is important to reduce the number of raw matches. Second, there are many repeated structures in the hyperspectral vessel images, enlarging the number of false matches. If all these outliers were input into the transformational model, sub-optimal results might be obtained. Therefore, we proposed a reliable method to kick out most of the outliers using relevant information of multiple bands.

Suppose {x_i_} and {y_j_} ∈ R^2^ are the sets of feature points of band i and j, Γ_ij_ is the estimated transform between the two bands. Consider rigid transform here for simplicity, and then the mapping between the two sets could be represented as,

$$y_{j}=\Gamma_{ij}x_{i}+r_{ij}=R_{ij}x_{i}+T_{ij}+r_{ij}$$ (3)

where *R*_*ij*_ is a 2 × 2 rotation matrix and *T*_*ij*_ is a 2 × 1 translation vector, *r*_*ij*_ is the residual errors. The inliers can then be defined as those with residual errors less than predefined threshold, while the outliers are above the threshold,

$$\Gamma_{ij}=\left\{ \begin{array}{l} \;inlier\;if\;\|r_{ij}\|\leq \varepsilon \\ outlier\;if\;\|r_{ij}\|>\varepsilon \end{array}\right.$$ (4)

Now let {z_k_} be another set belonging to band k, Γ_jk_ denotes the estimated transform between band *j* and *k*,

$$z_{k}=\Gamma_{jk}y_{j}+r_{jk}=R_{jk}y_{j}+T_{jk}+r_{jk}$$ (5)

We can also map {*z*_*k*_} to band *i* and get its corresponding set $\left\{x_{i}^{'}\right\}$,

$$x_{i}^{'}=\Gamma_{ki}z_{k}+r_{ki}=R_{ki}z_{k}+T_{ki}+r_{ki}$$ (6)

Combining (3), (4) and (5), we can get,

$$x_{i}^{'}=R_{ki}R_{jk}R_{ij}x_{i}+R_{ki}R_{jk}T_{ij}+R_{ki}R_{jk}r_{ij}+R_{ki}T_{jk}+R_{ki}r_{jk}+T_{ki}+r_{ki}$$ (7)

Suppose all the mappings between bands i, j and k are exact and with zero residual errors, $\left\{x_{i}^{'}\right\}$ should be the same with the initial set {*x*_*i*_}. Thus the total residual error after the consecutive mappings from band i to j, j to k, and k back to i is,

$$r_{ii^{'}}=R_{ki}R_{jk}r_{ij}+R_{ki}r_{jk}+r_{ki}$$ (8)

If each of the mappings Γ_*ij*_, Γ_*jk*_ and Γ_*ki*_ is an inlier, which means, ‖*r*_*ij*_‖ ≤ *ε*, ‖*r*_*jk*_‖ ≤ *ε*, ‖*r*_*ki*_‖ ≤ *ε* then,

$$\|r_{ii^{'}}\|\leq \|R_{ki}R_{jk}r_{ij}\|+\|R_{ki}r_{jk}\|+\|r_{ki}\|\leq 3\varepsilon$$ (9)

as ‖*R*_*ki*_‖ = ‖*R*_*jk*_‖ = 1.

In Eq (9), the modulus is performed on the total residual error and each of the three parts is within the threshold ε, so that the summing of them is less than 3ε. We can conclude that after the consecutive mapping from band i to j, j to k, and k back to i, if each of the mapping is an inlier, the total residual errors should fall into a prescribed threshold. Thus we could kick out most of the outliers that are beyond the threshold. We could also increase the number of feature point sets by kicking out more outliers.

**Fig 2:**
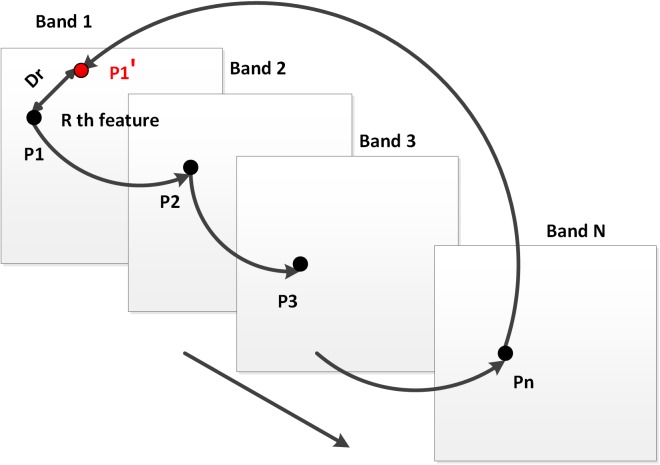
Diagram of kicking out outliers.

Fig 2 describes the discrimination of feature outliers from inliers. For every feature point shown as the black point named as P1 in band one, its corresponding match can be found in band two named as P2. Repeat this process from band to band until the feature point in the last band named as Pn find its match in the first band shown as the red point named as P1’. Then, after calculating the Euclidean distance between P1 and P1’ which is labeled as Dr in the figure, the feature point P1 would be identified as the outlier if Dr is larger than the given threshold.

Therefore, the proposed matching algorithm can be developed as follows:

Step1: Suppose that a scene of hyperspectral vessel image has N bands and the adjacent bands are put into feature detection part in pairwise. Then every single band has R feature descriptors, denoted as $Y_{r}^{n}$ (where n represents the nth band and r represents the rth feature).

Step 2: Based on the feature descriptors, the features are matched in pairs in the adjacent bands and are denoted as the R×R feature matrix Φ_n,n+1_. So that we get N feature matrixes, {Φ_1,2_, Φ_2,3_, Φ_3,4_, …, Φ_N,1_}.

Step3: For each of the R features, calculate the Euclidean distance between the first band and the last band, denoted as D_r_, using the information in the last feature matrix Φ_N,1_.

Step 4: If the distance of the rth feature is higher than the given threshold δ, the feature will be removed from the feature set.

### Oxygen saturation identification

After the registration scheme, the hyperspectral image was used for oxygen saturation analysis which was performed on the Matlab software platform (The Mathworks, Incorporated, Natick, Massachusetts). The fundamental method for measuring oxygen saturation in the microvasculature is to calculate oxygenated hemoglobin (O_2_Hb) and deoxygenated hemoglobin (HHb) based on Beer’s Law at two wavelengths λ_1_ and λ_2_ [3]. This dual-wavelength method determine the oxygen saturation by the ratio of O_2_Hb to the total hemoglobin concentration (sum of O_2_Hb and HHb) using the following equation:

$$S_{O_{2}}=\frac{C_{O_{2}Hb}}{C_{O_{2}Hb}+C_{HHb}}=\frac{\varepsilon_{HHb}^{\lambda_{1}}}{\left(\varepsilon_{O_{2}Hb}^{\lambda_{2}}-\varepsilon_{HHb}^{\lambda_{2}}\right)}*\frac{△A^{\lambda_{2}}}{△A^{\lambda_{1}}}-\frac{\varepsilon_{HHb}^{\lambda_{2}}}{\left(\varepsilon_{O_{2}Hb}^{\lambda_{2}}-\varepsilon_{HHb}^{\lambda_{2}}\right)}$$ (10)

where $C_{O_{2}Hb}$ and C_HHb_ are the concentrations of O_2_Hb and HHb, respectively, $\varepsilon_{O_{2}Hb}^{\lambda }$ and $\varepsilon_{HHb}^{\lambda }$ are the extinction coefficients for O_2_Hb and HHb at wavelength λ, △ A^λ^ is the absorbance at wavelength λ. If λ_1_ is set as the isobestic point, then the oxygen saturation maps can be obtained by calculating the ratio of the other wavelength expressed in Eq (10).

However, the dual-wavelength method is on the premise of known extinction coefficients. Therefore, for a more general oxygen saturation analysis without knowing extinction coefficients, the blind end-member and abundance extraction (BEAE) and quadratic blind linear unmixing (QBLU) algorithms are implemented on the hyperspectral vessel images. Detailed explanation can be found in [38] and the final end-member matrix is extracted according to Eq (11):

$$P^{N}=(I_{L}-\frac{1}{L}1_{L}1_{L}^{T})Y{\left(A^{N}\right)}^{T}{\left(A^{N}{\left(A^{N}\right)}^{T}+\rho O\right)}^{-1}+\frac{1}{L}1_{L}1_{N}^{T}$$ (11)

where $O=NI_{N}-1_{N}1_{N}^{T}$, Y represents the input data, A^N^ represents their correspondent abundances, ρ > 0 represents the regularization weight, N is the number of end member and L is the dimension number.

## Experiments and results

### Materials

The host animals we selected for DSFC preparation are nude mice weighing between 26g and 33g, provided by Animal Experiment Center in East China Normal University (ECNU) with the approval of the ethics committee of ECNU. Before the experiments, these mice were bred in the temperature of 26~28°C and humidity of 40~60% under aseptic condition. Then, ten adult (8–10 weeks of age) female BALB/c nu/nu mice were randomized into two groups: one is to use a normal window as a control (with no tumor implanted) and the other is to use a window with tumor (4T1 mammary adenocarcinoma cells) implanted. The ten mice were anesthetized (1mg/kg 3% pentobarbital) by intraperitoneal injection and placed on a sterile operating table. Pull the back skin of the mice by white light in order to find the area rich in blood vessels. Then, place a piece of window fixation plate in the selected area and make a mark on the hole of the screw. Punch with a diameter of less than 2 mm in the marked position, and carefully cut the hole in the same size of the screw with scissors. Make sure that the screws penetrate vertically into the holes to prevent damage or friction to the tissue of the mice. Next, place the second fixation plate on the other side of the mice's back skin and fix it by screws. Finally, place the coverslip on the cut skin and fix it with a fastening ring. Window chambers were installed into the mice’s skin by the principle of DSFC described in [6, 8], but there still some attentions need special stress. Firstly, select the observation area in which the skin has a rich vascularity. When implant the window, take caution in keeping the skin flat and smooth instead of wrinkled. Then, use sterilized scissor to remove the superficial skin layer without hurting blood vessels. Finally, complete the fixation by attaching titanium clips to the front frame and keep the environment as moist as possible throughout the data collection process.

### Image acquisition

After materials preparation (around 10am in the day), we use the home-made hyperspectral imaging system for image acquisition. The system consists of two parts, the Acousto-optic Tunable Filter (AOTF) based imaging hardware and the imaging process software. To begin with, the software is initialized by setting several parameters such as band numbers, timelapse, image format, etc. Then, the AOTF adapter is controlled by the software to filter the light ranging from 550 nm to 1000 nm. Thus, the Charge Coupled Device (CCD) detector is able to record the vessel image of each single mouse from two groups and transfer the data to the software for real-time display. The hyperspectral vessel image containing 60 bands is stored in the format of band sequential format (BSQ) with 1024×1024×12 bit per band.

### Image segmentation results

**Fig 3:**
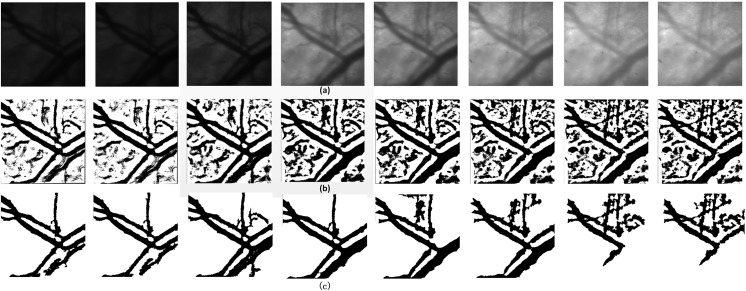
(a) single band images of hyperspectral vessel image, (b) binary images after fast threshold target extracting process, (c) target vessel after counter filtering process.

The hyperspectral vessel images reflect variance in light absorption among different wavelengths. However, the intensity differences among different bands usually make the procedure complicated during registration (As shown in Fig 3(A)). In this study, we proposed a fast and threshold level set method to segment the clear counter of target vessel. In the fast threshold target extracting part, the size was set to 50×50 pixels and corresponding binary images were obtained from the initial one. As illustrated in Fig 3(B), the target vessel was detected out of the tissue and distinct from the background, while it was still surrounded by numerous capillaries. After the counter filtering process, these noisy points were removed entirely so that clear and continuous blood vessel contours were recognizable in different bands(Fig 3(C)). Furthermore, the results also show that patterns of the extracted vessel counters vary with the band number and this difference is primarily caused by spectral features in different types of blood vessels. Specifically, this spectral feature could be explained by vasomotion and oxyhemoglobin change under different wavelength [13].

### Feature matching results

**Fig 4:**
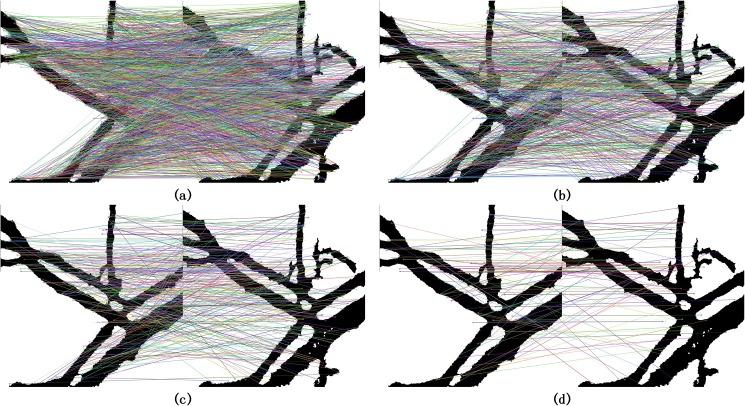
(a) Raw matches of initial method, (b) Raw matches of proposed method with 3 bands, (c) Raw matches of proposed method with 5 bands, (d) Raw matches of proposed method with 60 bands.

When the SURF method was performed on the segmented single band vessel image for feature detection and matching, it produced thousands of feature points of which most matches were outliers, as shown in Fig 4(A). These massive outliers not only decrease the matching accuracy but also increase the time consumption. Comparatively, Fig 4(B), 4(C) and 4(D) show that the proposed matching algorithm performs well in kicking out the outliers. It can be explained that the multiple bands of hyperspectral vessel image have close connection in shape and position transformation. Thus, when the mutual information was taken into consideration to set up the correspondence within images, the redundant feature points were reduced dramatically and outliers were removed as many as possible. Notice that the threshold ε is predetermined in negative correlation to the quality of the image datasets and the target accuracy. In terms of the general pixel shift in the hyperspectral blood images, 10 was used in this experiments. Table 1 compares the statistic data of raw matches (RM), correct matches (CM) in RM and accuracy between the initial SURF method and the proposed method. The initial SURF method refers to the matching method which only cares about feature descriptors instead of multiple band information. Moreover, we use the band numbers involved in the matching process to indicate the intrinsic distinction between the two methods. It can be seen from the table that the initial method processing two bands at a time detects 1020 correct matches out of 1951 raw matches and its accuracy is 52.3% which is lower than 68.9% that of the proposed method involving three bands. More importantly, when the proposed method increases the involved bands from 3 to 7, the raw matches has decreased sharply from 768 to 105 whereas the accuracy has risen significantly from 68.9% to 87.6%. The results are reasonable because most of the outliers are kicked out with more and more mutual information contained in the correspondence. Another superiority is that the raw matches do fast converge to a threshold and remain stable since the band numbers reach 7. However, the accuracy suffers a little decline to 84.3% when the total 60 bands are involved in the matching process, as shown in the right row of Table 1. It is probably caused by the image distortion in the last few bands due to the mice twitching with the ever-decreasing effect of anesthetic, but it still maintains its advantage.

**Table 1:** Comparison between the initial method and proposed method.

Measure	Initial method	Proposed method			
Band Numbers	2	3	5	7	60
RM	1951	768	309	105	102
CM	1020	529	249	92	86
Accuracy(%)	52.3	68.9	80.6	87.6	84.3

### Hyperspectral image registration results

**Fig 5:**
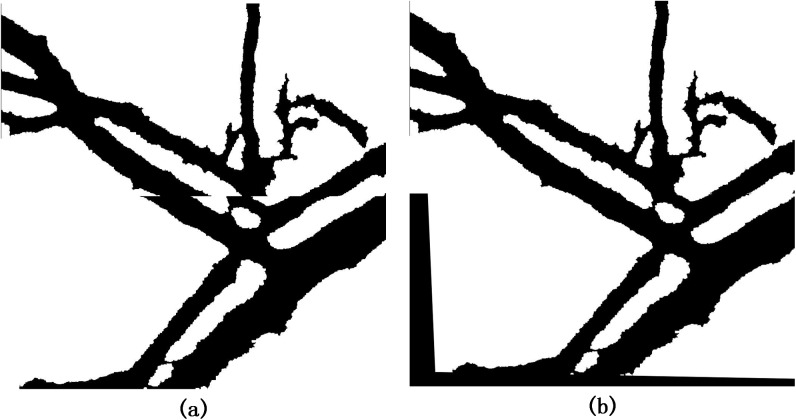
(a) splice of the 1st band and the 8th band, (b) the binary image of the 8th band after registration.

**Fig 6:**
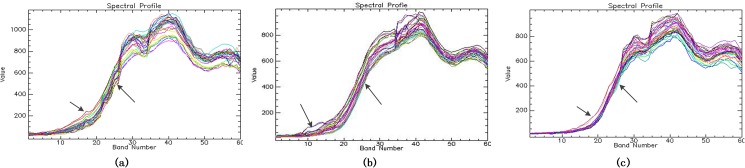
(a) spectrum of the hyperspctral vessel image before the registration, (b) after the initial registration, and (c) after the proposed registration.

Given the correlative matches of binary images of all bands, the proposed registration scheme is able to apply transformation to the hyperspectral vessel image. Fig 5(A) is the splicing of two uncorrected band images from which obvious dislocations can be seen. In contrast, the registrated binary image of the eighth band, shown in Fig 5(B), have eliminated these dislocations thoroughly. To make the enhancement visible, we select 36 points of a square region (6 pixels × 6 pixels) on the vessel to draw the spectra of these points. Then the collected spectra of these points from three images, the hyperspctral vessel image before registration, the hyperspectral vessel image after the initial and proposed registration are shown in Fig 6(A), 6(B) and 6(C) respectively. Theoretically, these adjacent points both belong to the vessel so that their spectra should remain consistent with each other as precise as possible. However, owing to the pixel shift, there would be an increase in the differences of these spectra which can be seen in Fig 6(A), inconsistent spectral values leading to scattered curves near the arrows. Whereas, after the registration, the pixel shift is repaired so that the differences of these spectra would decrease to a reasonable range. As shown in the upward arrows in Fig 6(B) and 6(C), the collected curves are more concentrated than those without registration. However, several curves’ spectral values that near the downward arrow extend the normal range (shown in Fig 6(B)), indicating that obvious mistakes still exist in the initial registration. In contrast, the proposed registration performs better in removing the incorrect matches. As a result, the arrows in Fig 6(C) show that the spectrum of the image after registration are smoother and more concentrated than that of the initial registration results. It can be explained that the proposed registration method takes both spectral and spatial information into consideration instead of only spatial features as the initial method does.

Moreover, we also analyze the registration error among the manual method, the initial method, and the proposed registration scheme. We use $\left(x_{r}^{n},y_{r}^{n}\right)$ to represent the coordinate of raw matches in the hyperspectral vessel image, where n donates the nth band and r donates the rth match. The error in X direction and Y direction can be calculate by the following equation:

$$E_{x}=\sqrt{\frac{1}{N}\sum_{i,j=1}^{N}\sum_{r=1}^{R}{\left(x_{i}^{r}-x_{j}^{r}\right)}^{2}}$$ (12)

$$E_{y}=\sqrt{\frac{1}{N}\sum_{i,j=1}^{N}\sum_{r=1}^{R}{\left(y_{i}^{r}-y_{j}^{r}\right)}^{2}}$$ (13)

where N donates the band number of the hyperspectral vessel image, R donates the number of feature points, i and j donate the adjacent bands. To evaluate the performance of the proposed registration scheme, the registration errors of the proposed method, the manual method, and initial method were calculated and compared. The error in X direction of the manual method, the initial method, and the proposed method is 2.03, 1.38, and 1.17, respectively. And in Y direction, the error is 1.98, 1.29, and 1.09, respectively. Thus, these data demonstrate that the proposed method outperforms the manual one by about one pixel both in X direction and Y direction. Meanwhile, the accuracy is improved by about 17% compared with the initial method.

### Oxygen saturation analysis

**Fig 7:**
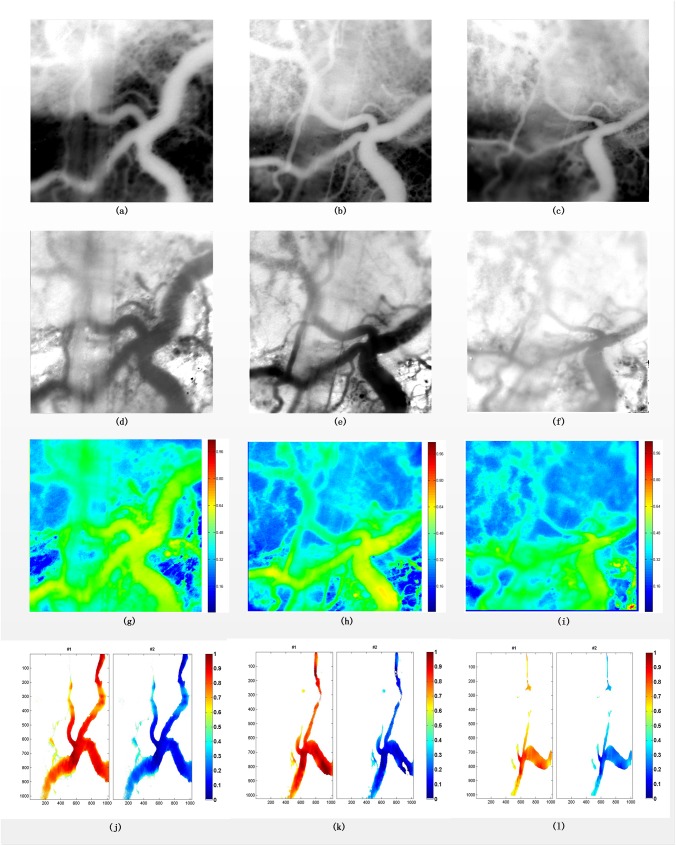
(a) the 12th band of hyperspectral image in the day 2, (b) the 12th band of hyperspectral image in the day 4, (c) the 12th band of hyperspectral image in the day 6, (d) the ratio image between 12th and 30th band in the day 2, (e) the ratio image between 12th and 30th band in the day 4, (f) the ratio image between 12th and 30th band in the day 6, (g) the pseudocolored maps in the day 2, (h) the pseudocolored maps in the day 4, (i) the pseudocolored maps in the day 6, (j) the abundance maps in the day 2, (k) the abundance maps in the day 4, (l) the abundance maps in the day 6.

After the registration, the hyperspectral vessel images are ready for the oxygen saturation analysis. To measure the change tendency of oxygen saturation in microvasculature, data were obtained from the mouse two days, four days, and six days after implantation of tumor as shown in the first, second and third column of Fig 7, respectively. We firstly test the dual-wavelength method in the extension of Beer’s Law and then, under the assumption of unknown molar extinction coefficient, the QBLU algorithm is applied to obtain abundance maps of microvasculature. As the isoabsorptive point is 573 nm in our experiment, the corresponding 12^th^ single band images are shown in the first row. Then the second row are the ratio images calculated by Eq (10) using the 12^th^ and 30^th^ band. From the pseudocolored maps in the third row, it can be seen that with the growth of tumor the oxygen saturation becomes lower and lower. This tendency is more significant in the abundance maps shown in the fourth row, which is also in accordance with the change of hemoglobin saturation reflecting that the vital signs of mice become decreasingly weak.

## Conclusion

In vivo imaging is a useful technique to observe and study blood oxygenation. With the installation of the dorsal skin fold window chamber (DSFC) model, many researchers have paid special attention to microvasculature oxygen saturation analysis. However, most of these relevant studies focus on the spatial information instead of spectral information. Hyperspectral images which contain abundant spatial and spectral information can produce better identification results than traditional methods. However, this technology induces the dislocation problem among multiple bands. Therefore, this paper proposed a registration scheme specific for in vivo hyperspectral vessel images. In the improved automatic registration scheme, the even and continuous vessel targets are obtained by determining a local threshold and constructing a level-set filter. Then, the SURF algorithm is applied to the binary images to analyze the feature points after eliminating the influence of intensity variation among multiple bands. Next, a reliable method based on correlative information among different bands is proposed to kicking out false matches. Finally, spectral analysis is used to evaluate the performance of hyperspectral vessel images registration. The experimental results show that the proposed scheme performs well as it can eliminate the influence of multiple intensity variation in feature detection and kicks out most of the outliers using relevant information of multiple bands in feature matching. Therefore, accurate spectra can be got from the registrated in vivo hyperspectral vessel image and then processed for the oxygen saturation analysis.

## Supporting information

S1 Text Original spectra data of vessel. (TXT)

S2 Text Spectra data of vessel after the initial registration. (TXT)

S3 Text Spectra data of vessel after the proposed registration. (TXT)
